# Piperazinediium tetra­chloridozincate(II)

**DOI:** 10.1107/S1600536809013981

**Published:** 2009-04-25

**Authors:** Pamela A. Sutherland, William T. A. Harrison

**Affiliations:** a Department of Chemistry, University of Aberdeen, Meston Walk, Aberdeen AB24 3UE, Scotland

## Abstract

In the title compound, (C_4_H_12_N_2_)[ZnCl_4_], the Zn atom adopts a slightly distorted tetra­hedral geometry. In the crystal, the dication and dianion inter­act by way of N—H⋯Cl and N—H⋯(Cl,Cl) hydrogen bonds to result in a layered network propagating in (010). The hydrogen-bonding network is unbalanced, with three Cl atoms accepting two hydrogen bonds each and one Cl atom not accepting any hydrogen bonds: the latter shows the shortest Zn—Cl bond length. The crystal studied was found to be an inversion twin.

## Related literature

For related structures, see: Bremner & Harrison (2003 ▶); Kefi & Nasr (2005 ▶); Wilkinson & Harrison (2007 ▶). For reference structural data, see: Allen *et al.* (1995 ▶). For details of graph-set theory, see: Bernstein *et al.* (1995 ▶).
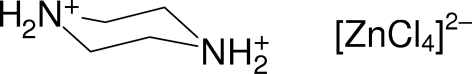


## Experimental

### 

#### Crystal data

(C_4_H_12_N_2_)[ZnCl_4_]
*M* *_r_* = 295.33
Orthorhombic, 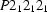

*a* = 8.2309 (3) Å
*b* = 11.0845 (3) Å
*c* = 11.8443 (4) Å
*V* = 1080.62 (6) Å^3^
*Z* = 4
Mo *K*α radiation
μ = 3.21 mm^−1^
*T* = 120 K
0.13 × 0.09 × 0.04 mm

#### Data collection

Nonius KappaCCD diffractometer
Absorption correction: multi-scan (*SADABS*; Bruker, 2003 ▶) *T*_min_ = 0.681, *T*_max_ = 0.882
8838 measured reflections
2388 independent reflections
2194 reflections with *I* > 2σ(*I*)
*R*_int_ = 0.058

#### Refinement

*R*[*F* ^2^ > 2σ(*F* ^2^)] = 0.042
*wR*(*F* ^2^) = 0.094
*S* = 1.08
2388 reflections
101 parameters
H-atom parameters constrained
Δρ_max_ = 0.92 e Å^−3^
Δρ_min_ = −0.77 e Å^−3^
Absolute structure: Flack (1983 ▶), 946 Friedel pairs
Flack parameter: 0.44 (2)

### 

Data collection: *COLLECT* (Nonius, 1998 ▶); cell refinement: *SCALEPACK* (Otwinowski & Minor, 1997 ▶); data reduction: *DENZO* (Otwinowski & Minor, 1997 ▶) and *SCALEPACK*, and *SORTAV* (Blessing, 1995 ▶); program(s) used to solve structure: *SHELXS97* (Sheldrick, 2008 ▶); program(s) used to refine structure: *SHELXL97* (Sheldrick, 2008 ▶); molecular graphics: *ORTEP-3* (Farrugia, 1997 ▶); software used to prepare material for publication: *SHELXL97*.

## Supplementary Material

Crystal structure: contains datablocks I, global. DOI: 10.1107/S1600536809013981/bg2253sup1.cif

Structure factors: contains datablocks I. DOI: 10.1107/S1600536809013981/bg2253Isup2.hkl

Additional supplementary materials:  crystallographic information; 3D view; checkCIF report

## Figures and Tables

**Table 1:** Selected bond lengths (Å)

Zn1—Cl1	2.2768 (12)
Zn1—Cl2	2.3119 (12)
Zn1—Cl3	2.2532 (12)
Zn1—Cl4	2.2634 (12)

**Table 2:** Hydrogen-bond geometry (Å, °)

*D*—H⋯*A*	*D*—H	H⋯*A*	*D*⋯*A*	*D*—H⋯*A*
N1—H1⋯Cl2	0.92	2.33	3.239 (5)	171
N1—H2⋯Cl4	0.92	2.77	3.168 (4)	107
N1—H2⋯Cl1^i^	0.92	2.49	3.206 (4)	135
N2—H3⋯Cl2^ii^	0.92	2.28	3.174 (4)	164
N2—H4⋯Cl4^iii^	0.92	2.50	3.194 (5)	133
N2—H4⋯Cl1^iii^	0.92	2.70	3.346 (4)	128

Symmetry codes: (i) 
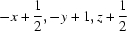
; (ii) 
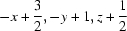
; (iii) 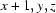.

**Fig. 1.**
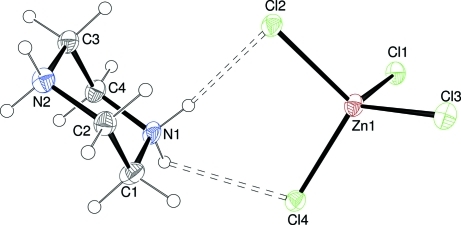
View of the molecular structure of (I) showing 50% displacement ellipsoids (arbitrary spheres for the H atoms) with the hydrogen bonds indicated by double dashed lines.

**Fig. 2.**
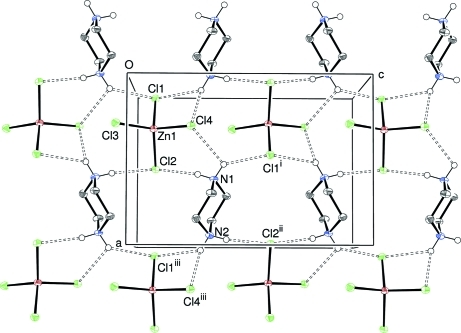
Part of an (010) hydrogen bonded sheet in the structure of (I) with the hydrogen bonds shown as double dashed lines. All the carbon-bound H atoms are omitted for clarity. Symmetry codes as in Table 2.

## supplementary crystallographic information

### Comment

As part of our ongoing investigations of hydrogen bonding networks in molecular salts containing metal-chlorido complexes, (Bremner & Harrison, 2003), we now report the structure of the title compound, (I). The structure of a monohydrate containing the same cation and anion was reported previously (Kefi & Nasr, 2005).

The Zn atom in (I) adopts a slightly distorted tetrahedral coordination arising from four chloride ions (Table 1, Fig. 1) and the organic dication adopts a typical chair geometry with normal bond lengths and angles (Allen *et al.*, 1995), the two nitrogen atoms being displaced from the mean plane of the four carbon atoms by -0.654 (7)Å and 0.685 (6)Å for N1 and N2, respectively.

In the crystal of (I), the components interact by way of simple N—H···Cl and bifurcated N—H···(Cl,Cl) hydrogen bonds (Table 2), such that each NH_2_ group forms one simple and one bifurcated bond. Some of the bifurcated H···Cl contacts are relatively long, but still significantly shorter than the H···Cl van der Waals' contact distance of 2.95 Å.

This hydrogen-bond connectivity results in a layered network propagating in (010) (Fig. 2). It is notable that this H bonding arrangement is unbalanced (Wilkinson & Harrison, 2007), with Cl1, Cl2 and Cl4 accepting two hydrogen bonds each, whereas Cl3 does not accept any H bonds. This may correlate with the fact that the Zn1—Cl3 bond length in (I) is the shortest of the four zinc–chloride links. Within the layers, various graph-set motifs (Bernstein *et al.*, 1995) are apparent, including *R*^2^_2_(6) and *R*^4^_4_(14) loops.

In (C_4_H_12_N_2_).[ZnCl_4_].H_2_O (Kefi & Nasr, 2005), a combination of N—H···Cl, N—H···O and O—H···Cl hydrogen bonds results in a three-dimensional network.

### Experimental

In an attempt to prepare a zinc–arsenite open-framework compound, ZnO, As_2_O_3_ and piperazine hexahydate were dissolved in a 1:1:1 molar ratio in dilute HCl solution. Colourless slabs of (I) grew as the water slowly evaporated, accompanied by octahedra of As_2_O_3_.

### Refinement

The H atoms were placed in idealized locations (C—H = 0.99 Å, N—H = 0.92 Å) and refined as riding with *U*_iso_(H) = 1.2*U*_eq_(carrier).

### Crystal data

**Table:**

(C_4_H_12_N_2_)[ZnCl_4_]	*F*(000) = 592
*M**_r_* = 295.33	*D*_x_ = 1.815 Mg m^−^^3^
Orthorhombic, *P*2_1_2_1_2_1_	Mo *K*α radiation, λ = 0.71073 Å
Hall symbol: P 2ac 2ab	Cell parameters from 8676 reflections
*a* = 8.2309 (3) Å	θ = 2.9–27.5°
*b* = 11.0845 (3) Å	µ = 3.21 mm^−^^1^
*c* = 11.8443 (4) Å	*T* = 120 K
*V* = 1080.62 (6) Å^3^	Slab, colourless
*Z* = 4	0.13 × 0.09 × 0.04 mm

### Data collection

**Table:**

Nonius KappaCCD diffractometer	2388 independent reflections
Radiation source: fine-focus sealed tube	2194 reflections with *I* > 2σ(*I*)
graphite	*R*_int_ = 0.058
ω and φ scans	θ_max_ = 27.5°, θ_min_ = 3.0°
Absorption correction: multi-scan (*SADABS*; Bruker, 2003)	*h* = −9→10
*T*_min_ = 0.681, *T*_max_ = 0.882	*k* = −12→14
8838 measured reflections	*l* = −15→15

### Refinement

**Table:**

Refinement on *F*^2^	Secondary atom site location: difference Fourier map
Least-squares matrix: full	Hydrogen site location: inferred from neighbouring sites
*R*[*F*^2^ > 2σ(*F*^2^)] = 0.042	H-atom parameters constrained
*wR*(*F*^2^) = 0.094	*w* = 1/[σ^2^(*F*_o_^2^) + (0.0219*P*)^2^ + 3.2663*P*] where *P* = (*F*_o_^2^ + 2*F*_c_^2^)/3
*S* = 1.08	(Δ/σ)_max_ < 0.001
2388 reflections	Δρ_max_ = 0.92 e Å^−^^3^
101 parameters	Δρ_min_ = −0.77 e Å^−^^3^
0 restraints	Absolute structure: Flack (1983), 946 Friedel pairs
Primary atom site location: structure-invariant direct methods	Flack parameter: 0.44 (2)

### Special details

**Table:**

Geometry. All e.s.d.'s (except the e.s.d. in the dihedral angle between two l.s. planes) are estimated using the full covariance matrix. The cell e.s.d.'s are taken into account individually in the estimation of e.s.d.'s in distances, angles and torsion angles; correlations between e.s.d.'s in cell parameters are only used when they are defined by crystal symmetry. An approximate (isotropic) treatment of cell e.s.d.'s is used for estimating e.s.d.'s involving l.s. planes.
Refinement. Refinement of *F*^2^ against ALL reflections. The weighted *R*-factor *wR* and goodness of fit *S* are based on *F*^2^, conventional *R*-factors *R* are based on *F*, with *F* set to zero for negative *F*^2^. The threshold expression of *F*^2^ > σ(*F*^2^) is used only for calculating *R*-factors(gt) *etc*. and is not relevant to the choice of reflections for refinement. *R*-factors based on *F*^2^ are statistically about twice as large as those based on *F*, and *R*- factors based on ALL data will be even larger.

### Fractional atomic coordinates and isotropic or equivalent isotropic displacement parameters (Å^2^)

**Table:**

	*x*	*y*	*z*	*U*_iso_*/*U*_eq_	
Zn1	0.27149 (6)	0.46334 (5)	0.09228 (5)	0.01746 (15)	
Cl1	0.06341 (13)	0.59864 (10)	0.09534 (11)	0.0192 (2)	
Cl2	0.51483 (13)	0.56726 (10)	0.09893 (11)	0.0193 (2)	
Cl3	0.24980 (15)	0.34993 (10)	−0.06487 (9)	0.0212 (3)	
Cl4	0.26367 (15)	0.35675 (9)	0.25519 (9)	0.0182 (3)	
C1	0.6765 (6)	0.3608 (4)	0.3437 (5)	0.0211 (11)	
H1A	0.6095	0.2999	0.3040	0.025*	
H1B	0.7022	0.3292	0.4198	0.025*	
C2	0.8319 (6)	0.3817 (4)	0.2790 (4)	0.0189 (10)	
H2A	0.8947	0.3057	0.2747	0.023*	
H2B	0.8062	0.4078	0.2010	0.023*	
C3	0.8383 (6)	0.5925 (5)	0.3449 (4)	0.0196 (10)	
H3A	0.8136	0.6227	0.2681	0.024*	
H3B	0.9052	0.6539	0.3839	0.024*	
C4	0.6815 (6)	0.5734 (4)	0.4092 (5)	0.0189 (9)	
H4A	0.7063	0.5505	0.4881	0.023*	
H4B	0.6185	0.6495	0.4106	0.023*	
N1	0.5828 (5)	0.4761 (4)	0.3544 (4)	0.0180 (9)	
H1	0.5509	0.5014	0.2839	0.022*	
H2	0.4907	0.4625	0.3966	0.022*	
N2	0.9310 (5)	0.4768 (4)	0.3369 (4)	0.0189 (9)	
H3	0.9583	0.4509	0.4082	0.023*	
H4	1.0256	0.4894	0.2972	0.023*	

### Atomic displacement parameters (Å^2^)

**Table:**

	*U*^11^	*U*^22^	*U*^33^	*U*^12^	*U*^13^	*U*^23^
Zn1	0.0130 (3)	0.0212 (3)	0.0182 (3)	−0.0007 (2)	−0.0003 (3)	−0.0015 (2)
Cl1	0.0164 (5)	0.0221 (5)	0.0191 (5)	0.0024 (4)	−0.0007 (5)	−0.0019 (5)
Cl2	0.0142 (5)	0.0253 (5)	0.0183 (5)	−0.0030 (4)	0.0008 (5)	0.0002 (5)
Cl3	0.0181 (6)	0.0240 (6)	0.0214 (6)	−0.0006 (5)	−0.0002 (5)	−0.0045 (4)
Cl4	0.0151 (6)	0.0188 (5)	0.0205 (5)	−0.0014 (5)	−0.0001 (5)	−0.0003 (4)
C1	0.016 (3)	0.017 (2)	0.030 (3)	−0.001 (2)	0.002 (2)	0.000 (2)
C2	0.019 (2)	0.019 (2)	0.019 (2)	0.0003 (19)	−0.001 (2)	−0.002 (2)
C3	0.013 (2)	0.021 (2)	0.024 (3)	0.001 (2)	0.000 (2)	0.001 (2)
C4	0.013 (2)	0.023 (2)	0.021 (2)	−0.0004 (17)	0.002 (2)	−0.002 (2)
N1	0.0091 (19)	0.025 (2)	0.020 (2)	−0.0019 (18)	0.0000 (16)	0.0001 (18)
N2	0.013 (2)	0.024 (2)	0.020 (2)	0.0030 (19)	0.0031 (17)	−0.0008 (18)

### Geometric parameters (Å, °)

**Table:**

Zn1—Cl1	2.2768 (12)	C3—N2	1.496 (6)
Zn1—Cl2	2.3119 (12)	C3—C4	1.513 (7)
Zn1—Cl3	2.2532 (12)	C3—H3A	0.9900
Zn1—Cl4	2.2634 (12)	C3—H3B	0.9900
C1—N1	1.499 (7)	C4—N1	1.498 (6)
C1—C2	1.510 (7)	C4—H4A	0.9900
C1—H1A	0.9900	C4—H4B	0.9900
C1—H1B	0.9900	N1—H1	0.9200
C2—N2	1.498 (6)	N1—H2	0.9200
C2—H2A	0.9900	N2—H3	0.9200
C2—H2B	0.9900	N2—H4	0.9200
			
Cl3—Zn1—Cl4	114.25 (4)	N2—C3—H3B	109.6
Cl3—Zn1—Cl1	108.73 (5)	C4—C3—H3B	109.6
Cl4—Zn1—Cl1	107.99 (5)	H3A—C3—H3B	108.1
Cl3—Zn1—Cl2	112.01 (5)	N1—C4—C3	110.2 (4)
Cl4—Zn1—Cl2	104.82 (5)	N1—C4—H4A	109.6
Cl1—Zn1—Cl2	108.84 (5)	C3—C4—H4A	109.6
N1—C1—C2	110.4 (4)	N1—C4—H4B	109.6
N1—C1—H1A	109.6	C3—C4—H4B	109.6
C2—C1—H1A	109.6	H4A—C4—H4B	108.1
N1—C1—H1B	109.6	C4—N1—C1	111.8 (4)
C2—C1—H1B	109.6	C4—N1—H1	109.2
H1A—C1—H1B	108.1	C1—N1—H1	109.2
N2—C2—C1	109.7 (4)	C4—N1—H2	109.2
N2—C2—H2A	109.7	C1—N1—H2	109.2
C1—C2—H2A	109.7	H1—N1—H2	107.9
N2—C2—H2B	109.7	C3—N2—C2	110.8 (4)
C1—C2—H2B	109.7	C3—N2—H3	109.5
H2A—C2—H2B	108.2	C2—N2—H3	109.5
N2—C3—C4	110.3 (4)	C3—N2—H4	109.5
N2—C3—H3A	109.6	C2—N2—H4	109.5
C4—C3—H3A	109.6	H3—N2—H4	108.1
			
N1—C1—C2—N2	57.4 (5)	C2—C1—N1—C4	−56.6 (5)
N2—C3—C4—N1	−56.4 (5)	C4—C3—N2—C2	58.8 (5)
C3—C4—N1—C1	55.8 (5)	C1—C2—N2—C3	−59.1 (5)

### Hydrogen-bond geometry (Å, °)

**Table:**

*D*—H···*A*	*D*—H	H···*A*	*D*···*A*	*D*—H···*A*
N1—H1···Cl2	0.92	2.33	3.239 (5)	171
N1—H2···Cl4	0.92	2.77	3.168 (4)	107
N1—H2···Cl1^i^	0.92	2.49	3.206 (4)	135
N2—H3···Cl2^ii^	0.92	2.28	3.174 (4)	164
N2—H4···Cl4^iii^	0.92	2.50	3.194 (5)	133
N2—H4···Cl1^iii^	0.92	2.70	3.346 (4)	128

Symmetry codes: (i) −*x*+1/2, −*y*+1, *z*+1/2; (ii) −*x*+3/2, −*y*+1, *z*+1/2; (iii) *x*+1, *y*, *z*.
